# Neurosustainability: A Scoping Review on the Neuro-Cognitive Bases of Sustainable Decision-Making

**DOI:** 10.3390/brainsci15070678

**Published:** 2025-06-25

**Authors:** Letizia Richelli, Maria Arioli, Nicola Canessa

**Affiliations:** 1 IUSS Cognitive Neuroscience (ICON) Center, Scuola Universitaria Superiore IUSS, 27100 Pavia, Italy; letizia.richelli@iusspavia.it; 2 Istituti Clinici Scientifici Maugeri IRCCS, Cognitive Neuroscience Laboratory of Pavia Institute, 27100 Pavia, Italy; 3 Department of Human and Social Sciences, University of Bergamo, 24129 Bergamo, Italy; maria.arioli@unibg.it

**Keywords:** sustainable decision-making, pro-environmental behaviors, climate change, interventions, eco-health, sustainability psychology, environmental psychology

## Abstract

As climate change continues to endanger a sustainable global condition, a growing literature investigates how to pursue green practices to fight its effects. Individuals are the essential starting point for such bottom-up attempts, with their attitudes towards sustainability driving pro-environmental behaviors (PEBs). **Objectives**: Based on the available relevant literature, this scoping review aims to delve into the processes underlying people’s sustainable decision-making (SDM) associated with PEBs. **Methods**: A scientific literature search was performed through (a) an active database search and (b) the identification of studies via reference and citation tracking. Results were screened and selected in Rayyan. **Results**: Included articles (*n* = 30) heterogeneously reported cognitive and neural aspects of SDM shaping PEBs. These proved to (a) recruit brain areas involved in mentalizing and moral cognition (likely because of their role in processing the interplay between personal and contextual factors rather than moral considerations in themselves); (b) undergo the same modulatory influences shaping other kinds of prosocial/cooperative behaviors; and (c) include brain areas involved in attentional/monitoring and emotional/motivational processes, alongside those consistently associated with decision-making processes. **Conclusions**: These results help interpret the available evidence on the neuro-cognitive bases of SDM while focusing on potential interventions to foster better practices and mitigate the adverse repercussions of climate change on human and global health.

## 1. Introduction

### 1.1. Conceptual Background

The latest disruptions in climate change patterns have been closely examined due to their impact on the planet and human well-being, both physical and mental [1]. Climatic impacts pose a concrete threat to physical health by negatively affecting respiratory and cardiovascular systems, in turn favoring mortality and infectious diseases [2]. Mental health is also endangered, both directly by disruptions connected to extreme weather events (increasing the rate of depression and post-traumatic stress disorder) and indirectly by real or perceived effects on society and one’s own future (leading to psychological and emotional distress, referred to as “eco-anxiety”) [3]. The increasing concerns raised by the association between environmental conditions and human well-being have been addressed by an interdisciplinary research field known as “eco-health”, aiming to promote sustainable and equitable health outcomes for both humans and the broader ecosystem. In this work, we consider neuroscience and cognitive psychology as promising approaches for achieving this goal. Cognitive neuroscience can contribute to this endeavor by studying the environment’s impact on brain plasticity and functioning, both in terms of the negative effects of climate change [4] and of the positive effects of exposure to nature [5]. By taking advantage of a wealth of evidence on human biases in judgment and decision-making, cognitive neuroscience can inform policymakers and research on the development of experimental settings, interventions and evaluation protocols ultimately aimed at nudging human behaviors towards a more harmonious relationship with the environment [6].

As climate change has become of central concern across natural and social sciences, an inter- and trans-disciplinary approach is the most suitable to comprehensively understand the issue and to develop effective long-term mitigation and adaptation strategies to overcome it [7,8]. For these purposes, the concepts of Sustainability and Sustainable Development have been widely employed to convey a broader meaning beyond mere environmentalism [9]. Sustainability is defined as the ability to meet present needs while safeguarding the ability of future generations to meet their own needs [10]. Sustainable Development, on the other hand, represents the guiding path to achieve this goal. Their principles include both social and economic dimensions of the environmental problem, whose solutions have been identified into 17 Sustainable Development Goals encompassing the economic, social, and biosphere aspects of climate change that should be addressed to achieve sustainability [11]. Undoubtedly, addressing the complexity and globality of this phenomenon requires radical changes in both the social and economic systems that have created and fueled the environmental problem.

With this scoping review, we focus on the decisional processes revolving around the concept of sustainable behaviors and address the underlying cognitive and neural mechanisms of Sustainable Decision-Making (SDM), i.e., decision-making processes involved in performing behaviors aimed to avoid harm and/or generate positive impacts on the economy, society, and environment. These behaviors are implemented with the intention of ensuring the well-being of both present and future generations, while also addressing current needs. We expect that gaining a deeper understanding about the neuro-cognitive bases of SDM can provide novel insights into the precursors of environmentally conscious behaviors at the individual and societal levels, as well as inform the development of effective strategies contributing to a more sustainable future for our planet [12,13,14].

### 1.2. Sustainable Behaviors

Alongside top-down solutions, such as eco-friendly policies and reforms, pursuing sustainable goals also requires bottom-up efforts by single individuals. While individual actions may have limited direct environmental impact, they are fundamental not only because societies’ ability to adapt to climate change is linked to their ability to act collectively [15], but also for their potential in influencing reforms, educating others, and increasing awareness [16,17]. However, building knowledge of practices in communities alone is not sufficient, if not sustained by strategies conveying the self-efficacy that is required to implement them. Simply communicating the risks of climate change is not always effective, as it might not translate into actual behavioral change. This is likely because threat information tends to be more prominent than efficacy information, resulting in unwanted defensive reactions (e.g., rejection, psychological distance, resistance to change), and climate change risks are often more abstractly than concretely represented [18], causing a discrepancy between what people perceive about the risks and what the actual risks are [19].

A starting point, in this respect, is facilitating and supporting SDM processes underlying sustainable behaviors, or pro-environmental behaviors (PEBs). SDM represents a crucial interface between ecological attitudes and environmental action. Therefore, it is important to adequately unveil the decision-making aspects favoring PEBs and their modulating variables. While the whole range of “sustainable” actions is virtually included in the PEBs label [20,21], the complexity and variety of influences on their implementation prevents a thorough description of the underlying SDM processes. To date, it is commonly acknowledged that PEBs are shaped by external, interpersonal, and intrapersonal determinants such as, respectively, costs and availability of resources, social norms and influences, self-efficacy and attitudes [22,23,24]. Much less is known, instead, on the cognitive and neural processes underlying SDM and on the variables modulating their connection with actual PEBs, as investigated by the rising field of “Neurosustainability” [25]. Importantly, we refer to “Neurosustainability” as a set of neural pathways underlying SDM [25], as opposed to the “sustainability” of neurons and their plasticity [26,27].

### 1.3. Sustainable Decision-Making, So Far

Performing targeted research on SDM can be challenging due to its complexity. Nevertheless, valuable insights are provided by related active fields such as neuroeconomics, neuromarketing, and consumer neuroscience.

Neuroeconomics combines principles from neuroscience, economics and psychology to investigate the neural mechanisms underlying economic decision-making [28] and provides insights on the reasons why people make (un)sustainable choices. Significant progress has been made since Simon’s [29] concept of bounded rationality, according to which individuals make satisfactory—rather than optimal—decisions due to temporal and cognitive limitations. Kahneman and Tversky’s [30] Prospect Theory addressed such gaps in terms of heuristics, i.e., mental shortcuts overcoming these limitations, that, however, entail biased judgments and choices. Because of intrinsic features of sustainability issues, some of these biases are particularly relevant to SDM [31,32,33], such as experiential vagueness, long-term effects, complexity and uncertainty, threat of the status quo, threat of social status, personal vs. community interest, and group pressure [34]. For instance, people often prioritize personal interests over PEBs that could benefit the broader community, and this tendency directly impacts SDM, in which the cognitive frame of gains and losses can be shifted from an individual to a more comprehensive perspective. SDM typically involves a trade-off between the personal investment of resources to behave sustainably [35] and the broader impact of one’s actions influencing an entire common resource such as the environment [36]. As such, environmental outcomes involve high stakes alongside wide spatial and temporal dimensions, as they encompass considerations regarding society and a large temporal horizon. However, individuals are usually unaware of the direct outcomes of their actions on the environment and may exhibit immediate aversion to personal losses, such as costs outweighing benefits, but not necessarily to environmental losses. Hence, SDM is characterized by a situation of great uncertainty which can negatively affect people’s willingness to behave sustainably. As a result, individuals often apply to their relationship with the environment the same heuristics they would employ generally in complex decisions, thus adopting compensatory balancing heuristics such as “green beliefs”, i.e., “the misconceptions that green choices can compensate for unsustainable ones” [37,38] and moral licensing, i.e., “highlighting past pro-environmental behavior produces a “license” to engage in less pro-environmental behavior” [39,40].

Consumer science investigates the cognitive and neural processes underlying consumer behavior, particularly in terms of “attribute preference”, i.e., of the elements that consumers value the most when selecting products. The degree of sustainability of a product can also be included as a feature, and consumers’ environmental valuation may influence pursuing such product. At the same time, such consideration is influenced by specific determinants. For instance, when assigning a monetary value to environmental goods and services, individuals’ willingness to pay (WTP) is affected by context, as well as emotions, attitudes, and uncertainty around environmental outcomes [41]. However, it should come as no surprise that attitudes are more predictive of actual behavior “when personal costs are low or environmental benefits are high” [42].

To investigate in more depth the roots of these modulations, neuromarketing couples neuroimaging techniques (e.g., functional magnetic resonance imaging (fMRI), electroencephalography (EEG), functional near-infrared spectroscopy (fNIRS)), and neuromodulation techniques (e.g., transcranial direct current stimulation (tDCS) and transcranial magnetic stimulation (TMS)) with experimental paradigms tapping into consumers’ behavior and preferences and their susceptibility to target marketing, e.g., towards more sustainable choices [43,44,45]. Via studies on consumer preferences and “green” neuromarketing, this approach has provided several insights on some factors shaping SDM, such as WTP for renewable energy [46] and consumers’ disposition towards sustainable products. Specifically, sustainable labels were found to work as a likeability factor for products, both explicitly and implicitly [47,48,49]. This preference applies to fashion [50], everyday use products [51], food [52], and packaging [53], but might also depend on consumers’ individual characteristics, such as age [54] and ecological consciousness [55]. However, the preference for sustainable products does not necessarily translate into actual and effective sustainable choices and behaviors [56]. This misalignment between explicit preferences or beliefs regarding sustainable purchasing and actual choices/behaviors is defined as “attitude-behavioral gap” [57]. An example of it is the “sustainable fashion paradox” which refers to the explicit claim of support to ethical and socially responsible fashion industries by individuals who are not, however, willing to pay more for their products when cheaper versions are made available, e.g., by socially unresponsible ones [50,58]. This gap constitutes an incentive to so-called “greenwashing”, where companies make consumers believe that (1) they are sustainable while they are not, and (2) they are selling them a cheap, yet sustainable product, when it is not. In this case, the decision not to buy consumer goods may be the authentic sustainable act [59]. Finally, even when people report to conduct PEBs, a mismatch between self-reported PEBs and actual behaviors might occur due to social desirability bias or lack of awareness about the impact of one’s own behavior [60,61]. For these reasons, and because priming some products over others and influencing consumers’ choices in favor of sustainability might raise ethical concerns [62], the contribution of cognitive neuroscience is needed to identify and understand the neural bases of SDM and its modulating factors, to increase the chances of PEBs rather than neuromarketing effectiveness [63].

On this ground, we reviewed the available evidence on the neuro-cognitive correlates of sustainable attitudes and behaviors, under the hypothesis that they are closely related to the neural bases of ethical self-regulation and moral cognition [59,64,65] as well as prosocial decision-making [66,67]. Expected correlates of SDM might include brain structures previously associated with decision-making per se, and particularly the dorsolateral prefrontal areas associated with executive functions such as working memory, planning and self-control [68]. Fronto-limbic structures such as the ventromedial prefrontal cortex, playing a key role in emotional processing and emotion-based decision-making [69,70], might drive individuals to make sustainable decisions out of an empathic disposition, an ethic of care for others and the environment, concern for future generations, or the desire for a harmonious relationship with nature [71]. To the best of our knowledge, this is the first attempt to identify and synthesize, via a scoping review, the main trajectories of the available emerging evidence on the neuro-cognitive bases of SDM.

## 2. Methods

The Preferred Reporting Items for Systematic Reviews and Meta-Analyses extension for Scoping Reviews (PRISMA-ScR) checklist [72] guided the scientific search for this work (more details are provided in Supplementary Material) via (1) active scientific literature searches and (2) identification of studies via other methods (e.g., results from additional searches such as backward and forward “snowballing” searches consisting of, respectively, reference and citation tracking).

Active scientific literature searches were conducted in 4 relevant databases (i.e., ScienceDirect, PubMed, EBSCOhost, and Scopus) in October 2024. No specific year range up to the target year, subject population criteria (i.e., age or health status) or minimum sample size were applied. Grey literature was not considered. Only published, peer-reviewed articles written in English, involving human participants, and addressing the cognitive and neural bases of sustainable decision-making were included. To prevent an over-extended pool of results, we used selected keywords from an initial extensive pool. Indeed, there is no consensus among scholars on specific terms that relate to the focus of this review, while many are used interchangeably. More specifically, the keywords “sustainability|sustainable|climate change|green|environment|eco|ecology|eco-friendly” were considered for the environmental aspects under investigation, and ultimately only “sustainab*” was selected. The keywords “neuro|neural|neuroscience|brain|cognitive|cognition|IAT|fMRI|EEG|tDCS|Stroop” were considered for exploring the cognitive and neural aspects of the research, and ultimately “neur*|cognit*” were selected. The keywords “reasoning|thinking|judgement|heuristic|bias|decision making|decision-making” were considered for exploring the decision-making processes under investigation, and ultimately only “decision making” and “decision-making” were selected. These terms were searched in titles and abstracts, as well as keywords when allowed by the advanced search option of the database website. The search string was consistent across databases but adapted to the specifics of each search engine design. For instance, on PubMed the search string looked like the following:

(sustainab* [Title/Abstract]) AND (neur* [Title/Abstract] OR cogniti* [Title/Abstract]) AND (decision making [Title/Abstract] OR decision-making [Title/Abstract])

Each reference list was screened (based on the title within the database) and imported in Rayyan [73], an online and free tool for systematic literature review, which automatically removes duplicates and systematically collects articles’ information (e.g., the article abstract). In Rayyan, retrieved results were initially labelled as “include”, “exclude”, “maybe” based on their abstract until there were no “undecided” titles left.

Additional studies were found by (1) cross-referencing included articles that had already been full-text screened (i.e., backward snowballing or reference tracking) and (2) checking how many other studies had referenced those included papers (i.e., forward snowballing or citation tracking).

## 3. Results

The aforementioned keywords led to identifying 844 records (*n* = 192 from PubMed; *n* = 84 from EBSCOhost; *n* = 268 from ScienceDirect; and *n* = 300 from Scopus). Among these records, 234 were detected as duplicate by Rayyan, and 579 were excluded for other reasons (i.e., even though these papers addressed topics connected to the concepts of sustainability, or decision-making, or neuroscience, or cognition, they did not focus on the cognitive and neural bases of sustainable decision-making). The remaining 31 abstracts were screened, and 16 were sought for retrieval (the others being labelled as “excluded”). All these short-listed articles were retrieved and assessed for eligibility, and six were excluded, as they did not inform on the cognitive and/or neural aspects of sustainable decision-making, as evaluated first by the three authors independently in Rayyan and discussed in case of disagreements (with tallied votes from the three raters when needed).

Among the articles detected via other methods, 20 were identified via reference or citation tracking, sought for retrieval, retrieved and assessed for eligibility. None was excluded, as evaluated first by the three authors independently in Rayyan and discussed in case of disagreements (with tallied votes from the three raters when needed).

**Figure 1:**
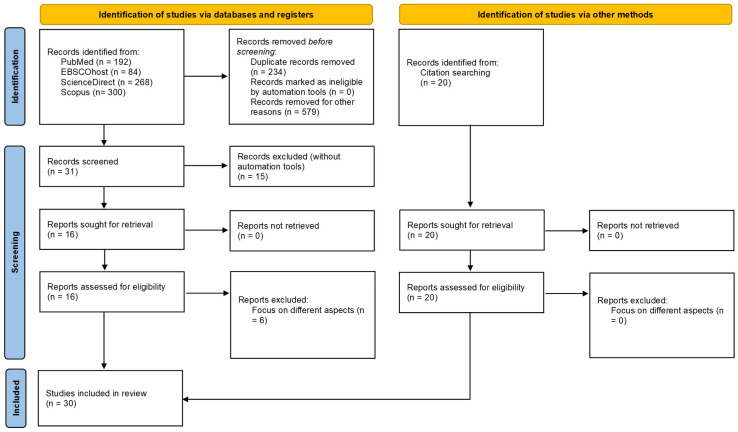
PRISMA flow diagram. From: [97]. For more information, visit: http://www.prisma-statement.org.

As shown in Figure 1, the whole selection process resulted in a final pool of 30 studies [6,13,14,47,52,58,71,74,75,76,77,78,79,80,81,82,83,84,85,86,87,88,89,90,91,92,93,94,95,96], a summary of which is provided in Table 1.

## 4. Discussion

**Figure 2:**
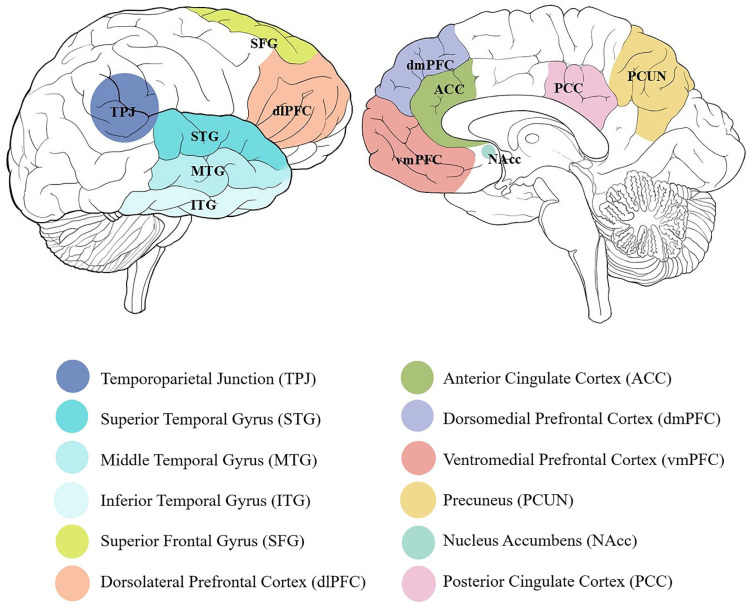
Brain areas involved in sustainable decision-making: TPJ [71,74,83], STG [78], MTG [78,79], ITG [78,79], SFG [52,79,92], dlPFC [74,75,87], ACC [52,58,74,92], dmPFC [71,74,78,79,81,87,92], vmPFC [52,76], PCUN [71], NAcc [89], PCC [52,71].

We summarized the available evidence on the cognitive and neural bases of SDM to better understand the processes enabling, and even promoting, sustainable choices. The present scoping review of the available scientific literature highlighted multiple cognitive processes and brain structures that appear to underpin SDM (see Figure 2 for an overview of the brain areas involved). In particular, the presence of recurrent topics within the included studies supported this first attempt to classify both the cognitive and neural bases of SDM. Namely, making decisions concerning the environment and sustainability was found to engage brain structures that have previously and independently been associated with (a) mentalizing and moral cognition, (b) cooperation and/or prosocial behavior, (c) reward processing and motivation, and their modulation on attentional processes. As SDM engages cognitive and neural mechanisms similar to those involved in prosocial and moral decision-making, we propose that these aspects contribute meaningfully to SDM. However, SDM encompasses a broader framework, including sustainable behaviors that go beyond the scope of prosocial behavior (actions intended to benefit others) and moral behavior (actions driven by a sense of moral obligation). We will first discuss these three trajectories within the rising field of neurosustainability, to highlight the cognitive processes potentially contributing to SDM and their neural bases, and then suggest potential implications of these findings in terms of addressing the implementation of PEBs and framing intervention strategies that foster sustainable development.

### 4.1. Moral Behavior and Mentalizing

As predicted by the Cognitive Elaboration Model of Ethical Decision-Making [98], individuals’ propensity to make sustainable decisions may vary depending more on the moral intensity of the situation, i.e., the perceived moral imperative in an ethical decision-making situation, rather than the characteristics of the decision-maker. Because morality seems to contribute to shaping a disposition towards sustainability, this relationship has been investigated under the hypothesis that moral attitude represents an intrinsic motivator to SDM, promoting a sense of fairness and shaping social norms [77,78]. In this respect, one study assessed managers and finance students in moral maturity, perceived moral intensity around environmental issues, and attitudes towards the environment [77], under the hypothesis that higher moral and ethical development would reflect in greater inclination towards SDM. Despite no connection between moral development and attention to sustainability, a significant relationship was found between the characteristics of environmental issues and people’s willingness to act more responsibly [77]. These findings suggested that a sustainable attitude is explained mainly by the interaction between the individuals’ personal characteristics and contextual factors rather than being directly shaped by moral considerations.

To unveil the neural bases of SDM and their potential modulation by moral considerations, ROIs activations from an fMRI study on sustainable consumer choices were compared with those that the Neurosynth toolbox associates with moral behavior, mentalizing, empathy, and emotion processing [78]. This approach highlighted activations specific to choosing sustainable (vs. non-sustainable) products in areas previously associated with mentalizing (dmPFC and left middle/inferior temporal gyrus) and moral cognition (dmPFC and left superior/middle temporal gyrus), thereby supporting the notion that SDM involves neural activity related to high-level cognitive inferences related to mentalizing and moral decisions. Similar results have already been reported in another study in which choosing sustainable (vs. non-sustainable) products was reported to engage regions associated with social cognition, self-referential thoughts, and mentalizing, such as the dmPFC and the left inferior/middle temporal gyrus [79]. Based on the available evidence on their functional role, these activations likely reflect people’s awareness of what others might think of their choices, highlighting the significant role that the interpersonal component plays in SDM, probably due to the social implications of such decisions [79]. Moreover, prosocial orientation and perspective-taking are known to moderate self-conscious emotions [99]. Placing high value on others’ welfare or adopting their viewpoints reflects in higher rates of sustainable actions (when pride is anticipated from taking responsible action such as contributing to environmental protection) and lower rates of harmful actions (when shame is anticipated from moral transgressions such as harming ecological systems) [100]. This suggests that an empathic disposition, as well as an ethic of care for others and the environment as an extension of oneself, fosters internalized moral standards that guide SDM through emotional accountability [59,64]. This capacity to anticipate how we might feel following certain actions, i.e., affective forecasting, eventually influences the implementation of morally charged behaviors, highlighting the role of anticipated and experienced emotions in connecting moral and prosocial aspects of SDM [101].

### 4.2. Prosocial and Cooperative Behaviors

Based on the societal implications of individual SDM, the affective and social counterpart of sustainable behavior has been extensively studied through economic games such as the several variants of the commons dilemma task (i.e., a dilemma task in which the participant decides how much to take from a common resource; [91]). The Fish Game is a popular instance of this family of tasks, requiring multiple participants to manage a limited amount of shared environmental resources, and, therefore, the trade-off between personal and social/intergenerational interests [102]. The game-theoretical principles underlying these tasks embody inherent features of intergenerational sustainability dilemmas, such as the temporal delay between actions and consequences, the social and temporal gap between benefactors and beneficiaries, non-reciprocity, and unidirectionality [74,87]. Studies using these experimental paradigms reported participants’ tendency towards sustainable behavior to depend on one’s intrinsic—rather than extrinsic—value orientations, and to be fostered whenever a relatedness-emphasizing prime (i.e., cooperation-related words) was provided before the task [86]. The tendency to engage in cooperative and sustainable behavior seems also to be influenced by being aware of others’ actions. While this modulation might be expected to result in emulative attitudes, there is evidence that—when facing another’s unsustainable and competitive behavior—people tend to sacrifice their own interests for a better environmental outcome, outweighing others’ greed with stronger pro-environmental values [90]. This might also be facilitated by people’s perception and reference shifting from a personal/individualistic to a more collective and environmentally conscious frame [55]. For instance, there is fMRI evidence that exposure to climate change cues reflects in decreased activation of areas associated with self-referential processing, such as the dmPFC, PCUN and PCC, and increased activation of occipito-temporal areas, possibly underlying attentional shifting to external stimuli [71].

Prosocial attitudes associated with pro-environmental behavioral outcomes seem to be reflected in distinctive patterns of brain structure and function that appear to favor SDM, especially involving distinct parts of the prefrontal cortex, such as right lateral prefrontal areas underlying cognitive control [75]. In particular, the disposition towards sustainable choices relates to greater cortical thickness of both the dlPFC and dmPFC [87]. In line with this neuro-structural evidence, stronger functional connectivity between and within networks involving the ACC/dlPFC and TPJ/dmPFC correlates with individuals’ dispositions towards sustainable choices and actions [74]. These regions are indeed known to underpin important aspects of SDM. The ACC is a key node of the fronto-limbic system associated both with attentional control and the monitoring of cognitive and emotional processes [103], connecting with dlPFC to enable self-control and override spontaneous selfish drives [104]. On the other hand, TPJ and dmPFC are crucial nodes of the mentalizing network [105], likely underpinning its contribution to higher-order processes interfacing with social cognition. Available data from other research lines suggests that the dmPFC is involved in evaluating the consequences of one’s own choices on others [106], possibly by incorporating their perspective, particularly when they are considered dissimilar to us, as in the case of future, thus socially distant, generations. This view of the possible functional role of dmPFC in SDM fits with recent meta-analytic evidence that this region underpins social cognitive processing not only by “representing” another’s mental states, but also by detecting the extent to which they align with one’s own ones (i.e., relational, rather than representational, processing) [107,108,109]; see also [110].

### 4.3. Delayed Reward and Prospective Thinking

While much can be already inferred on the modulation of SDM by multiple drivers of prosocial behavior such as fairness, cooperation and personal values [94], their contribution is strictly intertwined with evaluating, anticipating, and managing the inherent motivations in processing immediate vs. delayed rewards and punishments, all shaping individual attitudes and their expression into actual behaviors [111].

There is, nowadays, extensive evidence that the activity of the reward circuitry—and particularly of its key node in the ventral striatum—provides reliable metrics of people’s implicit attitudes predicting their actual behaviors [112]. Preliminary fMRI results indicate that this conclusion might hold also in the case of environmental attitudes and sustainable behaviors. For instance, eco-labels signaling fair trade products were found to increase activations in the NAcc—a key node of the ventral striatum associated with gain anticipation and positive affect [89]—alongside ACC, PCC and superior frontal gyrus [52]. Moreover, they appear to evoke smaller P2 and N2 amplitudes, possibly reflecting a positive preliminary evaluation of affective content and decreased conflict monitoring [47]. These findings strengthen the hypothesis that consumers evaluate positively the “sustainability” attribute and are, in principle, more willing to pay for it, even when the product itself is more hedonic than strictly utilitarian [95]. However, when participants were asked to evaluate the pleasantness and perceived sustainability of different types of advertisements (i.e., promoting sustainable products vs. control ones), a gap was found between self-reported scores favoring “green” advertisements and fMRI data showing ventral striatal and ventromedial prefrontal activations in association with the processing of control—rather than sustainable—advertisements [93]. Based on the putative direct relationship between the likelihood of approach behavior towards attended stimuli and the level of activation they induce in the reward pathway (e.g., [112]), these findings suggest that participants might favor sustainable products when expressing their attitudes and control products when engaged in actual purchasing [93]. Seemingly contradictory findings [89,93] can be reconciled by considering the context and modality of sustainable cues, i.e., explicit eco-labels embedded in concrete consumer choices (where affective valuation directly preceded purchasing) vs. abstract environmental messaging prompting moral self-evaluation rather than immediate reward anticipation. For this reason, when discussing the role of motivation and reward processing in behavioral change, it is also necessary to consider the role of the temporal delay at which rewards are expected to be achieved [80]. For environmental conservation behaviors, the benefits associated with investing in sustainable products and technologies often become tangible only after a long time. Longstanding evidence on so-called “delay discounting” [113], however, shows a spontaneous human preference for behaviors resulting in immediate rewards, in a sort of iteration of those survival instincts that have characterized the human species since the beginning [114]. With the aim of nudging individuals beyond immediate rewards to promote long-term goals, some studies have assessed the effect of (a) boosting the value of an abstract, long-term “green” claim of a product by expressing it as a concrete, short-term benefit [96] and (b) promoting prospective thinking as a means for long-lasting behavioral change [76]. In the latter study, participants were asked to verbally report how feasible it would be for them to increase sustainable/decrease unsustainable behaviors in their everyday life, while attending to related images during fMRI scanning. The observed association between the willingness to adopt more sustainable behaviors and the engagement of both the vmPFC and the hippocampus was suggested to underpin new mental representations of past behavioral routines. Conversely, prospectively thinking of no longer engaging in environmentally harmful actions recruited the dlPFC, a key node of the executive control network in charge of impulse control [104], which suggested the involvement of inhibitory mechanisms acting upon representations of unsustainable behaviors [76]. However, this is not the only neural pattern distinguishing between mental representations of sustainable and unsustainable behaviors. Further distinctions revolve around the role of episodic memory, wherein the perception and emotional evaluation of positive and negative environment-related mental images appear to engage distinct brain mechanisms. Namely, envisioning images depicting non-pleasant vs. pleasant and non-beautiful vs. beautiful environments recruits areas underlying top-down cognitive control (superior frontal gyrus and inferior frontal gyrus bilaterally), alongside regions underlying executive demands of working memory (left superior frontal gyrus) and emotion processing (the ACC and mPFC projecting to the amygdala) [92].

Overall, these results suggest that processing a negative environment, compared with a positive one, entails a more demanding management of trade-offs involving affective/deliberative, impulsive/controlled and personal/social trade-offs, in turn requiring complex benefit/cost analyses, emotional regulation, and motor control to override prepotent responses.

### 4.4. Applications and Interventions

The multi-disciplinary effort towards feasible ways for a more sustainable future goes from proposing solutions directly aimed at fostering individuals’ PEB (e.g., by addressing the whole complexity of behavioral determinants of SDM) to supporting research fields that address their precursors and drivers (i.e., by diversifying methods and integrating disciplines and practices) [115]. Cognitive neuroscience represents a potentially powerful tool in this endeavor, providing valuable insights on the drivers of behavior change, neuroforecasting and predicting society-level SDM, as well as bottom-up guiding policies [6,88]. This scoping review shows that characterizing the cognitive and neural determinants of SDM allows researchers to envision novel research lines assessing their modulating factors and their susceptibility to interventions aimed to increase the tendency towards sustainable attitudes and PEBs.

#### 4.4.1. Priming

Various interventions have been proposed to reduce the gap between explicit pro-environmental statements and actual sustainable behavior. One such attempt is grounded in the use of priming techniques. These involve using one stimulus (e.g., semantic, perceptive, visual) to affect, often unconsciously, how individuals respond to another upcoming one. This relatively simple and low-cost intervention can shift both cognitive evaluations and neural processing by enhancing the salience and perceived value of sustainable features. For instance, a fNIRS study primed engineers to consider environmental and social sustainability when evaluating different design options, reporting that their increased positive beliefs in green infrastructure benefits were reflected by activations in vlPFC, dlPFC, and mPFC through different stages of decision-making [81]. Another study assessed whether the exposure to (vs. absence of) pro-environmental priming stimulus (e.g., video) differentially modulates brain responses to images of products with “green” logos indicating their sustainability vs. “control” images of products without such logos [58]. The expected priming effect, as indexed by higher sensitivity to the presence of the sustainable logo, reflected in stronger activity in the ACC, which in neuromarketing has been found to be associated with consumers’ positive responses to prosocial advertisement and with prosocial choices [116]. Based on these considerations, the ACC was suggested to enhance the sensitivity to, and awareness of, the sustainable characteristics of the products as conveyed by the sustainable logo. For research investigating SDM, increasing the salience of these features is a promising way to elicit reactions to environmental priming, which might then prompt in-depth reasoning on sustainable stimuli, thereby favoring a behavioral switch towards the consumption of more sustainable products [58].

#### 4.4.2. Neurostimulation Techniques

To better test causal inferences on SDM, studies have made use of techniques of neurostimulation, e.g., by applying anodal high-definition tDCS (HD-tDCS) to the right TPJ in participants engaged in a task tapping SDM [83]. Based on its role in mentalizing [117] and attentional switch [118], enhancing TPJ activity in association with an intergenerational fishing game was reported to increase the ability to consider (future) others’ needs when drawing from common pool resources, and thus to modulate one’s intergenerational sustainability [83]. Conversely, cathodal HD-tDCS has been applied to inhibit the left dlPFC in an SDM task, based on the notion that TMS-mediated disruption of the right dlPFC activity decreases self-control [119], to assess whether interfering with this region might affect participants’ tendency towards intergenerational sustainability [120]. In this case, reducing participants’ self-control caused them to make more sustainable choices in an SDM task [120], most likely because other regions involved in self-control account for participants’ empowerment to suppress unsustainable impulses [82]. Although neurostimulation techniques may not be practical for implementing SDM interventions in real-world settings, they remain valuable tools for establishing causal relationships between SDM correlates.

#### 4.4.3. Virtual Reality

Other attempts to investigate SDM have emphasized the potential of virtual reality (VR) to promote PEBs by enhancing cognitive engagement, emotional involvement, and response efficacy, alongside testing immersive settings, evaluative conditioning, and the salience of impact messages—even among children [121,122,123,124]. Specifically, multi-sensory simulation has been shown to make sustainability messages more salient and persuasive [121], in turn favoring implementation of PEBs beyond the laboratory context. For instance, interactive pop-ups displaying the environmental or health impacts of products were found to effectively boost beliefs on the efficacy of personal responses, leading to more pro-environmental food choices both during the VR experience and in real-life shopping up to two weeks later [122]. Moreover, VR was found to stimulate and sustain climate-related conversations more effectively than other immersive media formats, i.e., video or article, both immediately and over time [123]. This effect was reported to be mediated by emotional arousal and spatial presence, underscoring the value of VR in providing experiential learning [123]. To take full advantage of the potential of this tool, VR should be integrated and combined in neuroimaging and neurostimulation research to improve the typically low ecological validity of these methods and to best track real-time monitoring of emotional, cognitive engagement.

#### 4.4.4. Nudging

Outside a laboratory setting, “nudging” might be one of the most practical interventions to increase individuals’ PEBs. This notion refers to the subtle changes in the choice environment that leverage cognitive heuristics to influence behavior without restricting options, or significantly altering incentives, to steer individuals towards better, e.g., more sustainable, choices. Nudges can also reinforce individuals’ SDM by increasing awareness, communicating standards, providing relevant information and feedback, and highlighting choice options [84]. Typical examples include setting the more sustainable as the default option, using social comparison messages, informative eco-labels, or framing messages to emphasize benefits [20]. For instance, energy suppliers serving households and businesses in Switzerland implemented a green default policy, i.e., by providing energy from green sources unless otherwise requested, they shifted the uptake of green energy and made a significant difference in terms of emissions [125]. Even when the sustainable option was more expensive, this effect remained stable over several years [126].

Neuroeconomic research underscores how nudging offers policymakers a targeted, cost-effective intervention which can significantly improve individuals’ environmental impact by reshaping their SDM context without limiting freedom of choice [88]. Because nudging takes advantage of cognitive biases and may influence people without their awareness, it should be designed to be transparent, reversible, and aligned with the public interest, ensuring freedom of choice while advancing collective sustainability goals [62].

To conclude, insights from neuroscience studies and cognitive science not only inform our understanding of the cognitive and emotional mechanisms underpinning SDM, but also offer practical guidance for designing interventions and policies tailored to how individuals and populations actually make decisions [6,14]. Such applications can be successfully integrated into models of behavior change to develop adaptation strategies and support SDM, even on a larger or specific scale [13]. Following the results discussed here and considering the theory underlying behavior change intervention models (e.g., [22,127]), attempts to promote PEBs should be goal-primed, positively charged, appeal-increasing and emotion-driven to increase the likelihood for individuals to engage and shift their behavior towards a more sustainable lifestyle [84]. As people might be more likely to implement long-lasting behavioral adaptations when these are driven by psychological, cognitive, and cultural reinforcements (e.g., personal engagement, greater salience in personal goal setting, and greater intrinsically pleasurable salience), encouraging PEBs should involve a cultural priming perspective, therefore advancing sustainability management as a social movement [85]. Moreover, communications around climate change issues should be positively framed, emotionally engaging, customized, and culturally tailored. Rational narratives of facts and figures tend to desensitize receivers, while negative ones are coded as threatening stimuli by the anterior insula and amygdala, thereby bypassing controlled processing and making people resistant to further behavioral adaptations [14,85]. Finally, educating individuals, even from an early age (e.g., whether through formal education in schools or within the households), around the need for sustainable development and the moral implications of environmental harm—such as intergenerational justice, ecological responsibility, and global equity—can build a stronger internal motivation to act sustainably, which external cues and interventions can further support.

### 4.5. Limitations

First, our results are only up to date as of October 2024, and the included studies were more easily identified through alternative search methods than formal search in databases, which highlights the difficulty of developing a search string that can successfully encompass most studies related to such a nuanced subject. Indeed, this emerging topic is frequently referenced by many studies, particularly in neuromarketing or consumer preferences research, that do not use the term “sustainability” and do not necessarily explore it in depth. This consideration highlights the importance of adopting a unique and specific label for this field to make it more easily recognizable by future research. Moreover, due to methodological constraints, the results from studies investigating SDM in laboratory settings (e.g., exploring consumer preferences) suffer from limited ecological validity and do not always reflect the complexity of everyday sustainable choices, which are typically automatic, habitual, or emotionally driven, often without deliberate reflection on the sustainability of their actions [128]. Future research should explore the interaction between conscious and unconscious processes to examine how these findings might reshape the taxonomy of mechanisms underlying SDM and, whenever possible, take advantage of the combination of techniques enabling experimental control in ecologically valid settings, such as VR in conjunction with a cognitive and/or neural recording or stimulation technique. Additionally, the available data often concern selected population samples (typically undergraduate students) that may not account for the potentially confounding effect of factors such as age, ethnicity, or political orientation. To address the potential effect of these variables, future research should thus strive for balanced samples. Moreover, despite the fact that concerns about sustainability vary greatly by country, the current literature does not allow formal cross-countries comparisons yet.

## 5. Conclusions

This scoping review explored the available evidence on the factors modulating SDM, focusing both on its psychological and cognitive precursors and on their neural correlates. Valuable insights from fields like Neuroeconomics, Consumer Science, and Neuromarketing, along with studies grounded in the Game Theory framework, revealed recurrent common themes representing preliminary—but clearly identifiable—research trajectories. Growing evidence from this rising and highly interdisciplinary field indicates that SDM is a multifaceted process integrating inputs pertaining to moral cognition, mentalizing, social cooperation and prospective thinking, as well as “core” modulating factors of decision-making such as reward, emotion and attentional processing. Unveiling the relative weight of these factors in shaping individual differences in SDM certainly represents a major challenge for this field in the years to come. Still, a detailed characterization of the endogenous and exogenous precursors of SDM will help design interventions aimed both to ease behavioral adaptations to a fast-evolving context and to increase the chances of observing more sustainable attitudes and decisions to result in actual PEBs. While such interventions may in principle take various forms (e.g., priming or nudging), they cannot but consider these factors to foster individual SDM, thereby fueling a collective effort towards radically new ways to conceive economic utilities. For example, understanding the gap between individuals’ implicit and explicit attitudes towards sustainability, as well as their possible connection with mentalizing, socio-cognitive and moral skills, represents a key stage in this scientific endeavor. Although unravelling their role in shaping SDM is a complex challenge, the importance of its implications clearly shows that it is not one that can be avoided or postponed, and summarizing the evidence collected so far in this rising but lively research field represents a preliminary, and still essential, step towards the consolidation of SDM.

## Figures and Tables

**Table 1:** Panoramic of included studies.

Article	Title	Origin	Methodology	Study Aims and Purposes	Study Outcomes Related to Neuro-Cognitive SDM	Section
1. Baumgartner et al., 2023 [74]	Neural mechanisms underlying interindividual differences in intergenerational sustainable behavior	Switzerland	fMRI, with intergenerational sustainability dilemma game	To investigate whether individuals behave more sustainably when they present greater functional activity and connectivity within the cognitive control network and between this and the mentalizing network when making decisions affecting the next (vs. present) generation	Differences in neural communication within and between the mentalizing (TPJ/dmPFC) and cognitive control (ACC/dlPFC) networks are related to interindividual differences in intergenerational sustainable behavior. The stronger the functional connectivity within and between these networks during decision-making, the more individuals behaved intergenerationally sustainably. This suggests that differences in the engagement of perspective-taking and self-control processes underlie interindividual differences in intergenerational sustainable behavior.	Section 4.2
2. Baumgartner et al., 2019 [75]	Dissociable neural representations of future reward magnitude and delay during temporal discounting	Switzerland	EEG, with neural task-independent, ecologically valid assessment of everyday PEB	To identify interindividual markers that explain variance in the frequency of everyday PEB	The right PFC, an area involved in cognitive control and self-control processes, explains individual differences in PEB implementation. The higher the cortical baseline activation in this area, the higher the frequency of everyday PEB.	Section 4.2
3. Brevers et al., 2021 [76]	Brain mechanisms underlying prospective thinking of sustainable behaviors	Belgium	fMRI, cue-exposure paradigm	To explore the core network of brain regions involved in the prospective thinking about (un)sustainable behaviors	Increasing sustainable behaviors is perceived to be more feasible than reducing unsustainable ones. A stronger activation of the vmPFC and hippocampus is observed when picturing an increase in sustainable behaviors. Simulating the reduction of unsustainable behaviors triggers activation within the right dlPFC (associated with inhibitory-control processes), which is negatively associated with hippocampal activation (associated with memory). These findings suggest that the dlPFC downregulates brain regions that support memory retrieval of unsustainable behaviors. This mechanism could inhibit access to episodic details associated with unsustainable behaviors and allow prospective thinking of sustainable ones.	Section 4.3
4. Doell et al., 2023 [6]	Leveraging neuroscience for climate change research	Austria	Perspective article	To outline how neuroscientists can make substantial contributions to climate change research	Neuroscience can be used to investigate the negative impact of climate change on the human brain, identify ways to adapt, understand the neural substrates of decisions with pro-environmental and harmful outcomes, and provide neuroscience-based insights into communication and intervention strategies that aim to promote climate action.	Section 4.4
5. Eberhardt-Toth & Wasieleski, 2013 [77]	A cognitive elaboration model of sustainability decision making: Investigating financial managers’ orientation toward environmental issues	France	Research article (survey)	To examine individual-level cognitive factors associated with developing an orientation to sustainable development issues	The moral maturity of an individual and the perceived moral intensity of the sustainable issue are individual-level cognitive factors associated with developing an orientation to sustainable development issues among a population of French business practitioners.	Section 4.1
6. Enax et al., 2015 [52]	Effects of social sustainability signaling on neural valuation signals and taste-experience of food products	Germany	fMRI	To investigate neural and behavioral processes underlying the influence of fair-trade labeling on food valuation and choice	Labeling a product as sustainable increases activity in the ventral striatum, anterior and posterior cingulate, and in the superior frontal gyrus, regions important for reward-processing and salience. WTP for these products correlates with activity in the vmPFC. When a sustainable product is evaluated, the anterior cingulate, ventral striatum, and superior frontal gyrus exhibit task-related increases in functional connectivity to the vmPFC, revealing a highly probable directed modulation of the vmPFC by those three regions, suggesting a network which alters valuation processes.	Section 4.3
7. Goucher-Lambert et al., 2017 [78]	A meta-analytic approach for uncovering neural activation patterns of sustainable product preference decisions	USA	fMRI, with cross-comparison of ROIs with database of fMRI studies Neurosynth	To investigate multi-attribute preference judgments involving sustainability, to uncover differences in these judgments compared to those outside the context of sustainability	The activation of regions of interest (ROIs) associated with moral reasoning and mentalizing shows that these play an important role in evaluating (un)sustainable products.	Section 4.1
8. Goucher-Lambert et al., 2017 [79]	Inside the mind: using neuroimaging to understand moral product preference judgments involving sustainability	USA	fMRI	To investigate the neural processes behind multi-attribute product preference judgments for products for which the environmental impact is a known quantity	Including the environmental impact of a product affects preference for that product, i.e., functional attributes become more important and aesthetic attributes become less important when sustainability is a factor because of the mentalizing and moral reasoning processes involved.	Section 4.1
9. Hirsh et al., 2015 [80]	Analysis of delay discounting as a psychological measure of sustainable behavior	USA	Perspective article	To explore the relevance of delay discounting to issues of sustainability	Delay discounting, a process where individuals devalue outcomes based on their temporal delay, can serve as a powerful behavioral framework for understanding and influencing SDM.	Section 4.3
10. Hu & Shealy, 2022 [81]	Priming Engineers to Think About Sustainability: Cognitive and Neuro-Cognitive Evidence to Support the Adoption of Green Stormwater Design	USA	fNIRS	To test whether priming engineers to think about the environmental and social sustainability benefits of green infrastructure can influence what attributes engineers consider and how they weigh these attributes during the design decision-making process	Priming engineers to consider environmental and social sustainability before considering the cost and risk of each option increased both the perceived benefits they believed green infrastructure could provide and the likelihood they would recommend the green infrastructure option. Primed engineers exhibited lower oxyhemoglobin in their vlPFC, dlPFC, and mPFC through multiple phases of the judgment and decision-making process, suggesting that the priming intervention increases the cognitive representativeness or salience of the benefits for green infrastructure.	Section 4.2
11. Jin et al., 2018 [47]	Environmental-friendly eco-labeling matters: evidences from an ERPs Study	China	EEG	To investigate consumers’ attitudes toward eco-labeled food by comparing their neural processing of visual stimuli depicting eco-labeled and non-labeled food	Although participants claim to prefer buying sustainable food, smaller P2 and N2 amplitudes were found when pictures of sustainable food were presented. Amplitudes of P2 were negatively correlated with participants’ purchase intention, suggesting that, while the sustainable labeling was not to one’s own interests, it can still be evocative and induce consumers’ positive emotion, bringing less cognitive conflict to the purchase decision-making and resulting in a greater purchasing intention.	Section 4.3
12. Lalani et al., 2023 [13]	Systems Thinking in an era of climate change: Does cognitive neuroscience hold the key to improving environmental decision-making? A perspective on Climate-Smart Agriculture	United Kingdom (UK)	Perspective article	To explore System Thinking (ST) from a social science perspective, the cognitive neuroscience tools that could be used to explore ST abilities, the possible correlates of ST, the integration of different frameworks for understanding ST on a case study	Integrating ST with cognitive neuroscience, e.g., combining traditional concept mapping with neuroimaging tools like fNIRS, might help uncover hidden cognitive patterns and better understand how certain areas, e.g., the dlPFC and parietal cortex, and certain frameworks, e.g., observational learning, prospective thinking, and the theory of planned behavior, are involved in SDM. These insights can help enhance SDM for farmers in the Global South.	Section 4.4.4
13. Langenbach et al., 2019 [82]	Inhibition of the right dlPFC by theta burst stimulation does not alter sustainable decision-making	Switzerland	TMS	To disrupt the right dlPFC to provide causal evidence as to whether diminished self-control leads to less intergenerational sustainability	Inhibition of the right dlPFC, known to be involved in self-control, does not lead to less intergenerational sustainability.	Section 4.4.2
14. Langenbach et al., 2022 [83]	Mentalizing with the future: Electrical stimulation of the right TPJ increases sustainable decision-making	Switzerland	HD-tDCS, with behavioral economic paradigm	To test whether a lack of sustainability stems from insufficient intergenerational mentalizing	Excitation of the right TPJ, known to be involved in mentalizing, increases sustainable decision-making, while its inhibition has no effect.	Section 4.4.2
15. Lee et al., 2020 [58]	How to “Nudge” your consumers toward sustainable fashion consumption: An fMRI investigation	South Korea	fMRI	To explain how environmental priming can increase consumer preferences for fashion products with sustainable logos	Logos indicating sustainable products influence consumer preferences toward sustainable fashion and activate the ACC, involved in emotional and evaluative processing. Priming messages are more effective than direct interventions in boosting preference and activate the superior parietal lobule and bilateral lingual gyri associated with relational reasoning. Nudging consumers using subtle, emotionally resonant, cognitively engaging messages is more effective than overt sustainability campaigns in promoting sustainable choices.	Section 4.4.1
16. Leeuwis et al., 2022 [84]	A framework for application of consumer neuroscience in pro-environmental behavior change interventions	The Netherlands	Scoping review	To provide (1) a review of neuroscientific evidence for consumer attitude, behavior, and incentives-based SDM for behavior change interventions and (2) research directions to exploit the power of affective conditioning and neuroscience methods for promoting PEB engagement	Motivating behavior with reward or punishment will most likely get users engaged in climate change action via brain structures related to the reward system, such as the amygdala, NAcc, and PFC, where the reward information and subsequent affective responses are encoded. The intensity of the reward experience can be increased when the consumer is consciously considering the action to achieve it, making goal-directed behavior the potential aim of behavior change interventions.	Section 4.4.4
17. McDonald, 2018 [85]	Sustainability management: research insights from social cognitive neuroscience	New Zealand	Research article	To explore how insights from social cognitive neuroscience provide implications for challenges of sustainability management	Neuro-cognitive factors such as the amygdala activity, in-group/out-group differentiation, loss aversion, implicit persuasion and priming effects (shaped by both cognitive and cultural influences) affect sustainability management.	Section 4.4.4
18. Prentice & Sheldon 2015 [86]	Priming effects on cooperative behavior in social dilemmas: Considering the prime and the person	USA	Research article (priming with resources dilemma game)	To test whether people with a relatively more intrinsic vs. extrinsic value orientation are particularly likely to enact cooperative behavior in resource dilemmas when they are primed with relatedness goals	People with a high relative intrinsic vs. extrinsic value orientation are more likely to behave cooperatively when primed with relatedness goals. This effect only occurs when the prime suggests the potential for satisfying the relatedness goal, rather than merely referencing it, suggesting that primed goals only translate into behavior when they align with a person’s motivational values and appear rewarding.	Section 4.2
19. Rosales et al., 2022 [87]	Interindividual differences in intergenerational sustainable behavior are associated with cortical thickness of the dorsomedial and dorsolateral prefrontal cortex	Switzerland	fMRI, with intergenerational sustainability dilemma game	To look for objective, stable, and trait-like neural markers of interindividual differences in consequential intergenerational behavior	Individuals behaving sustainably are marked by greater cortical thickness of the dmPFC and dlPFC, areas involved in perspective-taking and self-control. Mediation analyses suggest that greater cortical thickness of these brain areas better enable individuals to take the perspective of future generations and to resist temptations to prioritize immediate personal benefits at the expense of future generations.	Section 4.2
20. Sawe, 2019 [88]	Adapting neuroeconomics for environmental and energy policy	USA	Perspective article	To articulate the potential of neuroeconomic methods to aid environmental policymakers interested in behavior change, i.e., in closing the energy efficiency gap	Combining neuroimaging with behavioral economics is a way to “neuroforecast”, i.e., forecast large-scale behavioral responses based on small-scale neural data, and can help policymakers understand how people process information, respond to eco-labels, make intertemporal trade-offs, and react to social norms and climate change messaging, ultimately enhancing the effectiveness of behavior-change interventions developments.	Section 4.4
21. Sawe et al., 2022 [89]	Neural responses clarify how ecolabels promote sustainable purchases	USA	fMRI	To examine how the eco-label influences choices of light bulbs within individuals, across individuals, and out-of-sample in a national survey	Logos that indicate sustainable products increase activity in neural regions like the NAcc, associated with positive affective responses. In more impulsive individuals, in particular, this neural activity reliably predicts individual purchasing decisions and, when averaged across subjects, also forecasts market-level demand in a separate national sample. This suggests that eco-labels can influence sustainable choices more through affective (emotional/reward-based) rather than deliberative (cognitive/calculative) SDM processes.	Section 4.3
22. Sawe & Chawla, 2021 [14]	Environmental neuroeconomics: how neuroscience can inform our understanding of human responses to climate change	USA	Perspective article	To explore neuroeconomic investigations of many factors relevant to climate change risk including affective response, processing of uncertainty, intertemporal choice, and social and cooperative decision-making	Insights from neuroeconomics, such as exploring how the brain processes affective responses, uncertainty, intertemporal tradeoffs, and social cooperation, can help predict both individual and collective behaviors. These insights have implications for climate policy, communication strategies, and welfare analysis, especially by highlighting differences in how people perceive risks, value the future, and engage in cooperative behavior.	Section 4.4
23. Sussman et al., 2016 [90]	Pro-environmental values matter in competitive but not cooperative commons dilemmas	USA	Research article (commons dilemma game)	To investigate whether the choice to conserve or be greedy in a commons dilemma may be influenced by the behavior of others and by pro-environmental values	In a competitive commons dilemma, individual with strong pro-environmental values tended to show significantly greater restraint compared to those with weak pro-environmental values. However, in cooperative situations, individuals’ pro-environmental values did not significantly affect behavior.	Section 4.2
24. Tarditi et al., 2020 [91]	Affective dilemmas: The impact of trait affect and state emotion on sustainable consumption decisions in a social dilemma task	Switzerland	Research article (social dilemma game)	To investigate the impact of trait affect and state emotion on individual consumption decisions in social dilemma tasks	Participants with high trait affect were more likely to reduce their consumption as resource scarcity increased, but only when the choice was presented in a gain frame. In participants with high trait affect, induced guilt led to reduced consumption in the gain frame, whereas induced pride led to increased investments in the loss frame.	Section 4.2
25. Vedder et al., 2015 [92]	Neurofunctional correlates of environmental cognition: an fMRI study with images from episodic memory	Germany	fMRI	To investigate how different environments affect people on a neural level	Visualizing non-pleasant, non-beautiful environments, relative to visualizing pleasant and beautiful ones, activated additional and more distributed brain regions, such as the left PFC and cortical midline structures.	Section 4.3
26. Vezich et al., 2016 [93]	The mere green effect: An fMRI study of pro-environmental advertisements	USA	fMRI	To understand why consumers’ self-reported preference of sustainable products is not reflected in purchase behaviors	Although participants claim to prefer sustainable ads over control ones, they present greater activation in regions associated with personal value and reward (vmPFC and ventral striatum) in response to control ads relative to sustainable ads, suggesting that this activity may be indexing a value signal computed during message exposure that may influence downstream purchase decisions, in contrast to self-reported evaluations that may reflect social desirability concerns absent at the point of purchase.	Section 4.3
27. Wang & van den Berg, 2021 [94]	Neuroscience and climate change: How brain recordings can help us understand human responses to climate change	The Netherlands	Review	To outline how carefully designed experiments that measure key neural processes, linked to specific cognitive processes, can provide powerful tools to answer research questions in climate change psychology	Neuroscience can provide insights beyond self-report methods, i.e., by uncovering implicit cognitive and emotional mechanisms, such as how people process fairness in climate policy, experience empathy toward future generations, or weigh self vs. other motivations in SDM. For this purpose, thoughtful integration with psychological theory and robust experimental design are necessary.	Section 4.2
28. Wei et al., 2023 [95]	Influence of utilitarian and hedonic attributes on willingness to pay green product premiums and neural mechanisms in China: an ERP study	China	EEG	To investigate how product attributes and premiums affect information processing, and thus SDM, by comparing consumers’ acceptance of hedonic and utilitarian sustainable products with different levels of premiums	Participants’ WTP for green premiums is higher for utilitarian products compared to hedonic products. Green premiums for utilitarian products elicit N4 component, indicating conflict and deeper cognitive evaluation, while green premiums for hedonic products elicit stronger P2 components, indicating greater early cognitive attention. This suggests that environmental justification can reduce the guilt associated with paying more for emotionally appealing products, and this mechanism can be used to leverage both the functional and emotional attributes of green products.	Section 4.3
29. Yin & Lee, 2023 [71]	Planet earth calling: unveiling the brain’s response to awe and driving eco-friendly consumption	South Korea	fMRI	To investigate the psychological and neural factors that can increase eco-friendly consumption	Climate messages decreased activation in self-referential areas (dmPFC, PCUN, PCC) and increased activation in external attention areas (occipitotemporal cortex, TPJ, ventral frontal cortex), suggesting a shift from self-focused to externally-focused processing.	Section 4.2
30. Zandstra et al., 2013 [96]	Understanding consumer decisions using behavioral economics	The Netherlands	Perspective article	To investigate how we can motivate consumers to resist the “now” and invest in their future, leading to sustainable or healthy habits	Framing delayed environmental benefits as immediate and relevant to the consumer can significantly increase the perceived value of sustainable claims and influence consumer SDM. This suggests that consumers are more likely to implement sustainable choices when long-term rewards are made emotionally and cognitively immediate.	Section 4.3

## Supplementary Materials

The following supporting information can be downloaded at: https://www.mdpi.com/article/10.3390/brainsci15070678/s1. Table S1: Preferred Reporting Items for Systematic Reviews and Meta-Analyses extension for Scoping Reviews (PRISMA-ScR) Checklist.

## Abbreviations

The following abbreviations are used in this manuscript:

(HD-)tDCS	(High-Definition) Transcranial Direct Current Stimulation
ACC	Anterior Cingulate Cortex
dlPFC	Dorsolateral Prefrontal Cortex
dmPFC	Dorsomedial Prefrontal Cortex
EEG	Electroencephalography
fMRI	Functional Magnetic Resonance Imaging
fNIRS	Functional Near-Infrared Spectroscopy
IAT	Implicit Association Test
ITG	Inferior Temporal Gyrus
MTG	Middle Temporal Gyrus
NAcc	Nucleus Accumbens
PCC	Posterior Cingulate Cortex
PCUN	Precuneus
PEBs	Pro-Environmental Behaviors
ROIs	Regions of Interest
SDM	Sustainable Decision-Making
SFG	Superior Frontal Gyrus
ST	System Thinking
STG	Superior Temporal Gyrus
TMS	Transcranial Magnetic Stimulation
TPJ	Temporoparietal Junction
vlPFC	Ventrolateral Prefrontal Cortex
vmPFC	Ventromedial Prefrontal Cortex
VR	Virtual Reality
WTP	Willingness to Pay
